# Insight into Potassium Vanadates as Visible-Light-Driven Photocatalysts: Synthesis of V(IV)-Rich Nano/Microstructures for the Photodegradation of Methylene Blue

**DOI:** 10.1021/acs.inorgchem.2c00136

**Published:** 2022-06-10

**Authors:** Małgorzata Nadolska, Mariusz Szkoda, Konrad Trzciński, Paweł Niedziałkowski, Jacek Ryl, Aleksandra Mielewczyk-Gryń, Karolina Górnicka, Marta Prześniak-Welenc

**Affiliations:** † Faculty of Applied Physics and Mathematics, Institute of Nanotechnology and Materials Engineering, Gdansk University of Technology, Narutowicza 11/12, Gdansk 80-233, Poland; ‡ Faculty of Chemistry, Gdansk University of Technology, Narutowicza11/12, Gdansk 80-233, Poland; § Faculty of Chemistry, University of Gdansk, Wita Stwosza 63, Gdansk 80-308, Poland

## Abstract

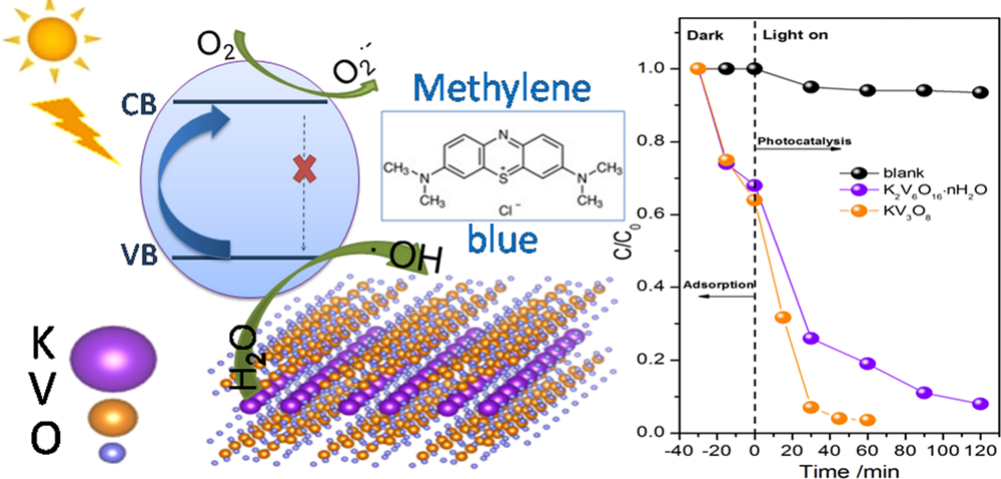

Photocatalysis is regarded as a promising tool for wastewater remediation. In recent years, many studies have focused on investigating novel photocatalysts driven by visible light. In this study, K_2_V_6_O_16_·*n*H_2_O nanobelts and KV_3_O_8_ microplatelets were synthesized and investigated as photocatalysts. Samples were obtained via the facile method based on liquid-phase exfoliation with ion exchange. By changing the synthesis temperature (20–80 °C), different compositions, morphologies, and V^4+^/V^5+^ ratios were obtained and investigated as photocatalysts for organic dye degradation. Potassium vanadates’ structural, morphological, and optical properties were characterized using X-ray diffraction(XRD), Fourier transform infrared spectroscopy (FTIR), X-ray photoelectron spectroscopy (XPS), Physical Property Measurement System (PPMS), thermogravimetric analysis (TGA) with mass spectrometry (MS), N_2_ adsorption, scanning electron microscopy (SEM), photoluminescence (PL), and UV–vis diffuse reflectance spectroscopy (DRS). Synthesized K_2_V_6_O_16_·*n*H_2_O and KV_3_O_8_ showed an efficient absorption in the visible wavelength region with a narrow band gap energy of 1.80 and 1.91 eV, respectively. Their photocatalytic activity was evaluated by the degradation of methylene blue (MB) under simulated solar light illumination. The KV_3_O_8_ microplatelets exhibited the greatest photocatalytic activity, resulting in more than 90% degradation of the dye within the first 30 min. It is suggested that the observed excellent photocatalytic performance is attributed to the high content of V^4+^ species. Furthermore, the influence of active species was investigated, and the mechanism responsible for the photodegradation of the MB dye was discussed for the first time for potassium vanadates.

## Abstract 2

Potassium vanadates were synthesized via the facile method based on liquid-phase exfoliation with ion exchange. Different synthesis temperatures (20−80 °C) resulted in various phase compositions, morphologies, and V^4+^/V^5+^ ratios. The obtained samples were demonstrated as efficient visible-light driven photocatalysts, resulting in more than 90% degradation of methylene blue within the first 30−60 min. The enhanced photoactivity is attributed to the high content of V^4+^ species, which are beneficial for the separation of photogenerated electrons and holes and their lifetime.

## Introduction

The search for new, efficient, and low-cost photocatalysts has attracted increasing attention due to their perfect utilization of clean and renewable solar energy for treating wastewaters. In the past years, various materials have been investigated and tested for water purification.^1−4^ Examples include metal^5,6^ and metal oxide nanoparticles,^7^ sulfides,^8,9^ nitrides,^10^ metal–organic frameworks,^11,12^ or carbon-based nanostructures.^13^ Recent studies demonstrate that metal vanadates exhibit promising visible-light photocatalytic reactivity in decomposing pollutants and water splitting.^14−17^ A series of compounds can be mentioned here, such as Ag_3_VO_4_,^18^ AgV_7_O_18_,^19^ CuV_2_O_6_,^20^ FeVO_4_,^21^ Cd_2_V_2_O_7_,^22^ InVO_4_,^23^ GdVO_4_,^24^ and BiVO_4_^25,26^ as the most known representatives from the vanadate family. The conducted research is mainly devoted to the nanostructured vanadates, which can appear in various forms such as nanoparticles,^27,28^ nanobelts,^29,30^ or nanotubes.^31^ It is well known that the shape and size play a significant role, as broadly described in the literature.^32−35^ The photocatalytic properties are also strongly dependent on the crystalline structure. Generally, the monoclinic scheelite BiVO_4_ (m-BiVO_4_) is considered to be more active than the other two tetragonal phases.^36−38^ In addition, the face-dependent photocatalytic behavior was also shown with the beneficial effect of (040) facets on contaminant degradation.^39−42^ Another critical factor in the photocatalytic reaction is crystal defects, for example, oxygen vacancies, which trap photogenerated pairs and reduce their recombination rate.^43^ The presence of oxygen vacancies can also broaden the activity range of a semiconductor oxide from UV to NIR light.^44^ As a result, new pathways to synthesize photocatalysts with controllable properties are sought.

In this study, we present a new synthesis method of potassium vanadates as efficient photocatalysts for methylene blue (MB) degradation. Potassium vanadates were prepared by the facile LPE-IonEx method (liquid-phase exfoliation with ion exchange), which was recently proposed by our group.^63^ LPE-IonEx is a straightforward, low-temperature, one-pot approach for the synthesis for transition metal oxide bronzes with controlled structural and morphological properties. Notably, the proposed method uses water as a solvent, making it eco-friendly. We demonstrated that, depending on the synthesis temperature (20–80 °C), the hydrated single-phase K_2_V_6_O_16_·*n*H_2_O to nonhydrated KV_3_O_8_ can be obtained through their mixture. In addition, an increase in temperature leads to increased V^4+^ concentration. The photocatalytic activity of prepared materials was evaluated by the degradation of an organic dye—methylene blue—in water under simulated solar light illumination. So far, KV_3_O_8_ has been considered as a precursor for V_3_O_7_ nanobelt synthesis for photocatalytic water splitting applications.^45^ To the best of our knowledge, the photocatalytic properties of nonhydrated phase KV_3_O_8_ have never been investigated. Furthermore, the mechanism and kinetics of photocatalytic degradation were studied for the first time.

## Experimental Section

### Synthesis

Potassium formate (99%, Sigma Aldrich) and V_2_O_5_ (99.2%, Alfa Aesar) without further purification were used as reagents. Milli-Q deionized water was used (resistivity >19 MΩ·cm). The samples were prepared via the LPE-IonEx method, where the procedure was as follows: 500 mg of V_2_O_5_ was added to 50 mL of a 1 M solution of potassium formate in deionized water. The mixture was vigorously stirred for 72 h, and the synthesis was conducted in four temperatures: 20, 40, 60, and 80 °C. The samples were labeled according to the reaction temperature as KVO-20, KVO-40, KVO-60, and KVO-80, respectively. From the obtained solution, rusty red to burnt orange to orange precipitations were collected by centrifugation. After washing several times with deionized water, products were dried overnight at 40 °C under reduced pressure (0.01 bar).

### Physicochemical Characterization

The X-ray diffractograms (XRD) were collected on a BrukerD2 Phaser diffractometer with Cu Kα radiation (λ = 1.5404 Å) in the range 2θ of 5–70° at room temperature. The samples’ morphology was studied at room temperature by an FEI Quanta FEG 250 scanning electron microscope (SEM) in secondary electron mode using an Everhart-Thornley detector (ETD). Fourier transform infrared (FTIR) spectra were measured on a Perkin Elmer Frontier spectrophotometer. The KBr pellet method was used, and transmittance spectra were recorded from 4000 to 500 cm^–1^ with a resolution of 4 cm^–1^. The high-resolution X-ray photoelectron spectroscopy (XPS) analysis was performed using an Escalab 250Xi device (ThermoFisher Scientific, USA) equipped with a monochromatic Al Kα source. Measurements were carried out at 25 eV pass energy with 0.05 eV energy step size. The X-ray spot size was 250 μm. The calibration of the XPS spectrum was done using the characteristic peak of adventitious carbon C 1s at 284.6 eV.^46^ Thermogravimetric analysis (TGA) was carried out under an argon atmosphere with a flow rate 60 mL min^–1^ in the temperature range of 40–400 °C (with a heating rate of 5 °C min^–1^) using Netzsch STA449 F1. A constant sample mass (20 ± 0.5 mg) was used. The thermal behavior has also been studied by EGA–MS (evolved gas analysis–MS). The gases that come out from the sample during heating were monitored by the quadruple mass spectrometer Netzsch QMS 403 Aëolos. The differential scanning calorimetry (DSC) measurement was performed under an argon atmosphere with a flow rate 60 mL min^–1^ in the temperature range of 35–450 °C (with a heating rate of 15 °C min^–1^) using a NETZSCH DSC 204 F1 Phoenix calorimeter.

Nitrogen adsorption–desorption isotherms were measured on a surface area analyzer (NOVAtouch 2, Quantachrome Instruments) at 77 K. Before the measurements, samples were degassed under a dynamic vacuum at 40 °C for 12 h. The specific surface area was calculated using the Brunauer–Emmett–Teller (BET) linear equation in the relative pressure range (*p*/*p*_0_) from 0.1 to 0.3. The correlation coefficient of the linear regression was not less than 0.99.

The UV–vis reflectance spectra of the selected materials were measured with a UV–vis spectrophotometer (Lambda 35, Perkin-Elmer) equipped with a diffuse reflectance accessory. The spectra were registered in a range of 300–900 nm with a scanning speed of 120 nm min^–1^. Band gap energy values were determined as the intercept of the tangent of the plot of transformation of the Kubelka–Munk function. To determine the energy band gap (*E*_bg_) of the chosen powders, the Kubelka–Munk function (eq 1) was applied:
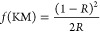
1where *R* is the reflectance.

The band gap was estimated by extrapolation of the linear region of (*f(*KM*) h*ν)^*n*^ vs *h*ν to *y* = 0, where the power ″*n*″ depends on the electron transition (*n* = 1/3, indirect forbidden (i.f.); *n* = 0.5, indirect allowed (i.a.); *n* = 2/3, direct forbidden (d.f.); and *n* = 2, direct allowed (d.a.)).^47^ The photoluminescence spectra were recorded using 0.3 m Czerny–Turner spectrograph (SR303i, Andor) equipped with an ICCD camera (DH740, Andor). Powders were excited with UV LED (365 nm, 9 nm FWHM, 350 mW).

### Magnetic Properties

Quantum Design Physical Property Measurement System (PPMS) with a vibrating sample magnetometer function was used to measure the temperature-dependent magnetic susceptibility (defined as χ = *M/H*, where *M* is the magnetization and *H* is the applied field). The temperature dependence of the zero-field cooled (ZFC) magnetization was measured in the applied field of 3 T. Magnetic measurements were performed on a sample of arbitrary shape with a mass of about 10 mg.

### Photocatalytic Activity

To evaluate the powders’ photocatalytic activity, the degradation rate of methylene blue (MB) dye was monitored. Twenty milligrams of catalysts and 50 mL of an aqueous solution of MB (*C*_0_ = 10^–5^ M) were transferred to the beaker. Before irradiation, the suspension was magnetically stirred in the dark for 30 min to achieve adsorption/desorption equilibrium. Then, the suspension was irradiated with sunlight irradiation (a high-pressure 150 W xenon lamp, LOT–Quantum Design GmbH equipped with an AM1.5G filter) with constant magnetic stirring (150 rpm). The intensity of the light that reached the solution’s surface was equal to 100 mW cm^–2^. The changes of the concentration of MB (*C*) during decomposition were monitored using a UV–vis spectrophotometer at a wavelength of 665 nm. The quantitative evaluation of the role of the chemical individuals responsible for dye decomposition was performed using appropriate scavengers (1 mM of *tert*-butyl alcohol, benzoquinone, and ammonium oxalate).

The MB products after photocatalytic degradation using KVO-20 and KVO-80 samples were analyzed by ultra-performance liquid chromatography quadrupole time-of-flight mass spectrometry (UPLC–QTOF-MS/MS). The analysis of solutions obtained after photocatalytic degradation was performed on an Agilent 1290 Infinity liquid chromatograph and Agilent 6550 iFunnel Q-TOF LC/MS System. The mobile phase consisted of phase A: 0. 1% aqueous formic acid with 0.3% acetonitrile and phase B: 0.1% formic acid in acetonitrile. The analysis was recorded with a gradient elution from 0 to 100% B during 10 min with an injection volume of 5 μL. The Agilent ZORBAX RRHD Eclipse Plus C18 column (95 Å, 2.1 × 50 mm, 1.8 μm) was used for the separation and detection of resulting products with a column temperature of 24 °C and flow rate 0.3 mL/min. The mass spectra were obtained in positive modes using the following operating parameters: capillary voltage 3.5 kV, nozzle voltage 2 kV, and fragmentor voltage 175 V. The gas temperature was 250 °C with a flow rate of 12 L/min and nebulizer pressure of 40 psi; the sheath gas temperature was 300 °C with a flow rate 11 L/min. The mass spectra were monitored in the range from 50 to 1000 *m*/*z* with a scan range of one spectrum per second. The obtained UPLC–QTOF-MS/MS data were analyzed using the Mass Hunter Qualitative Analysis software.

### Electrochemical Characterization

The electrochemical measurements were performed using an Ivium Vertex potentiostat/galvanostat in a three-electrode cell using Pt mesh as the counter electrode and Ag/AgCl (3 M KCl) as the reference electrode. The tested powder was deposited onto degreased FTO (fluorine-doped tin oxide) using a dip-coating method according to the procedure described previously.^48^ Measurements were performed in deaerated aqueous 0.2 M K_2_SO_4_. The Mott–Schottky analysis was performed to determine the flat band potential. The impedance spectra were recorded at a potential range from 0.4 to 0.9 V vs Ag/AgCl (3 M KCl). The potential was held before each spectrum registration to achieve a steady-state condition. The potential range for analysis was determined on the basis of a cyclic voltammetry (CV) curve. The space charge capacitance was determined from 1000 Hz frequency according to the formula *C*_sc_ = 1/ω*Z*″.

## Results and Discussion

### Structural Analysis

**Figure 1:**
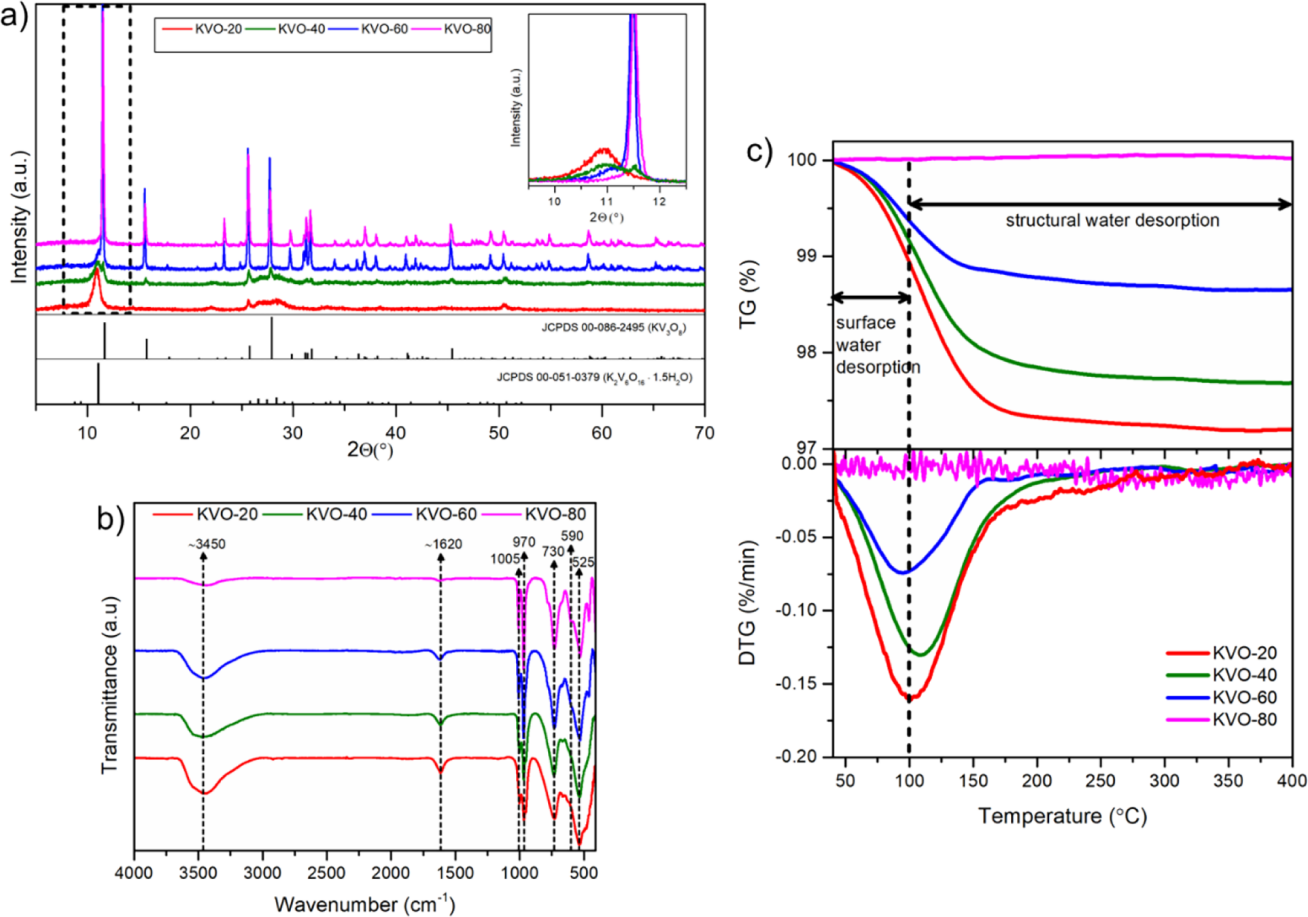
(a) XRD patterns, (b) FTIR spectra, and (c) TG and DTG curves of the samples obtained under different reaction temperatures. The inset shows the zoomed view of the XRD’s most intense peaks ascribed to KV_3_O_8_ (JCPDS card no. 00-086-24-95) and K_2_V_6_O_16_·1.5H_2_O (JCPDS card no. 00-051-0379).

The X-ray diffractograms were recorded to confirm the phase purity and crystallinity of obtained samples (see Figure1a). For the diffractogram for the sample synthesized at 20 °C (KVO-20), all indices can be indexed within hydrated potassium vanadate phase K_2_V_6_O_16_·1.5H_2_O, JCPDS card no. 00-051-0379, which corresponds to a monoclinic structure with lattice parameter values of *a* = 12.29 Å, *b* = 3.59 Å, and *c* = 16.01 Å. The main diffraction peak, located at approximately 11°, corresponds to the diffraction from the (002) crystallographic plane of this phase. Moreover, no signals of other phases were detected, indicating the high purity of the sample. For the samples synthesized at higher temperatures, additional peaks can be observed, indicating the presence of the secondary phase KV_3_O_8_, JCPDS card no. 00-086-24-95, which corresponds to a monoclinic structure with lattice parameter values of *a* = 4.97 Å, *b* = 8.38 Å, and *c* = 7.64 Å. The presence of KV_3_O_8_ is revealed by its characteristic diffraction peak located at approximately 11.5°, which corresponds to the (001) crystallographic plane (see inset in Figure 1a). With increasing synthesis temperature, the intensity of the diffraction peaks ascribed to the KV_3_O_8_ phase increases, and the first peak of KVO-40 and KVO-60 is split. This suggests the existence of two phases in these samples. The XRD pattern of sample KVO-80 shows indexed reflection only for the KV_3_O_8_ phase. The characteristic peak for the hydrated potassium vanadate phase is no more visible. FTIR spectra of the samples are shown in Figure 1b and summarized in Table S1. The bands at ∼1005 and ∼970 cm^–1^ can be assigned to V=O vibrations, while those at 525 and 590 cm^–1^ can be assigned to the symmetric and asymmetric stretching of V–O–V.^49^ The band at 730 cm^–1^ corresponds to bridging V–O···K stretching.^50^ In all spectra, the splitting of the V=O bands can be observed. Such phenomenon implies the distortions in the vanadium oxide layers and the existence of VO_5_ and VO_6_ polyhedra, which are typical for hexavanadates and mixed-valence vanadium compounds.^51,52^ Significant changes were also observed in the position of the absorption band located ca. 1005 cm^–1^. In comparison to KVO-20, the maximum for KVO-80 is shifted from 1000 to 1010 cm^–1^. This is linked to the shortening of the bond length and reducing the distance between vanadium oxide layers. The distance between layers for the hydrated potassium vanadate (K_2_V_6_O_16_·1.5H_2_O) is almost doubled than for the nonhydrated phase (KV_3_O_8_).^49,53,54^ Also, in all spectra, there are two extra bands at ∼3450 and ∼1620 cm^–1^ that can be associated with water molecules’ stretching and bending vibrations.^45^ Their positions vary within the samples, and peaks shift to the low wavenumber from 1633 and 3475 cm^–1^ for KVO-80 to 1615 and 3435 cm^–1^ for KVO-20. According to the literature, a decrease in wavenumber is evidence of the binding of water with other atoms.^55^ In case of anhydrous KV_3_O_8_ (KVO-80), physisorbed water molecules are only weakly bonded to the samples’ surface, while for hydrated K_2_V_6_O_16_·*n*H_2_O (KVO-20), water molecules are trapped in the crystalline lattice by either interactions with potassium ion or vanadium-oxide layers. Moreover, in the KVO-20, KVO-40, and KVO-60 spectra, an additional band at 950 cm^–1^ can be observed. The position of this band suggests the existence of the OH bridge between two metals, probably between V.^56^ Thermogravimetric measurements were performed to further investigate the weight percentage of crystalline water in samples (Figure 1c). According to the TG and DTG curves, the decomposition of the material occurred stepwise. Especially for single-phase, KVO-20 is seen on the DTG curve that a broad peak between 40 and 180 is asymmetric (which indicates two different decomposition kinetics) and overlaps with another broad one. Simultaneously, the molecular weight channels 17 (corresponding to OH) and 18 (H_2_O) on the EGA–MS curves showed a broad peak ion current corresponding to the shape of the peak on the DTG curve (Supporting Information Figure S1a). The ratio of the integrated ionic current of *m*/*z* = 17 and 18 is around 0.2 in the whole temperature range, which corresponds to a well-known signature of water molecules.^57^ Thus, the weight loss between 40 and 100 °C is attributed to the evaporation of physisorbed water and that between 100 and 400 °C to crystalline water.^58^ The weight loss of crystalline water for a single-phase hydrated sample (KVO-20) is 1.75 wt %, which corresponds to 0.65 molecule of water per K_2_V_6_O_16_ formula unit. The weight loss for KVO-40 and KVO-60 samples is smaller and equal to 1.48 and 0.72%, respectively. According to the XRD and FTIR results, these samples are composed of two phases: the hydrated and nonhydrated one. Therefore, upon comparison, the weight loss of the crystalline water in these samples will be smaller. The KVO-80 sample is thermally stable up to 350 °C, which is confirmed by DSC results where no thermal effects are visible (Supporting Information Figure S1b). The structural and thermal measurements confirmed that this sample consists of only a nonhydrated KV_3_O_8_ phase.

**Figure 2:**
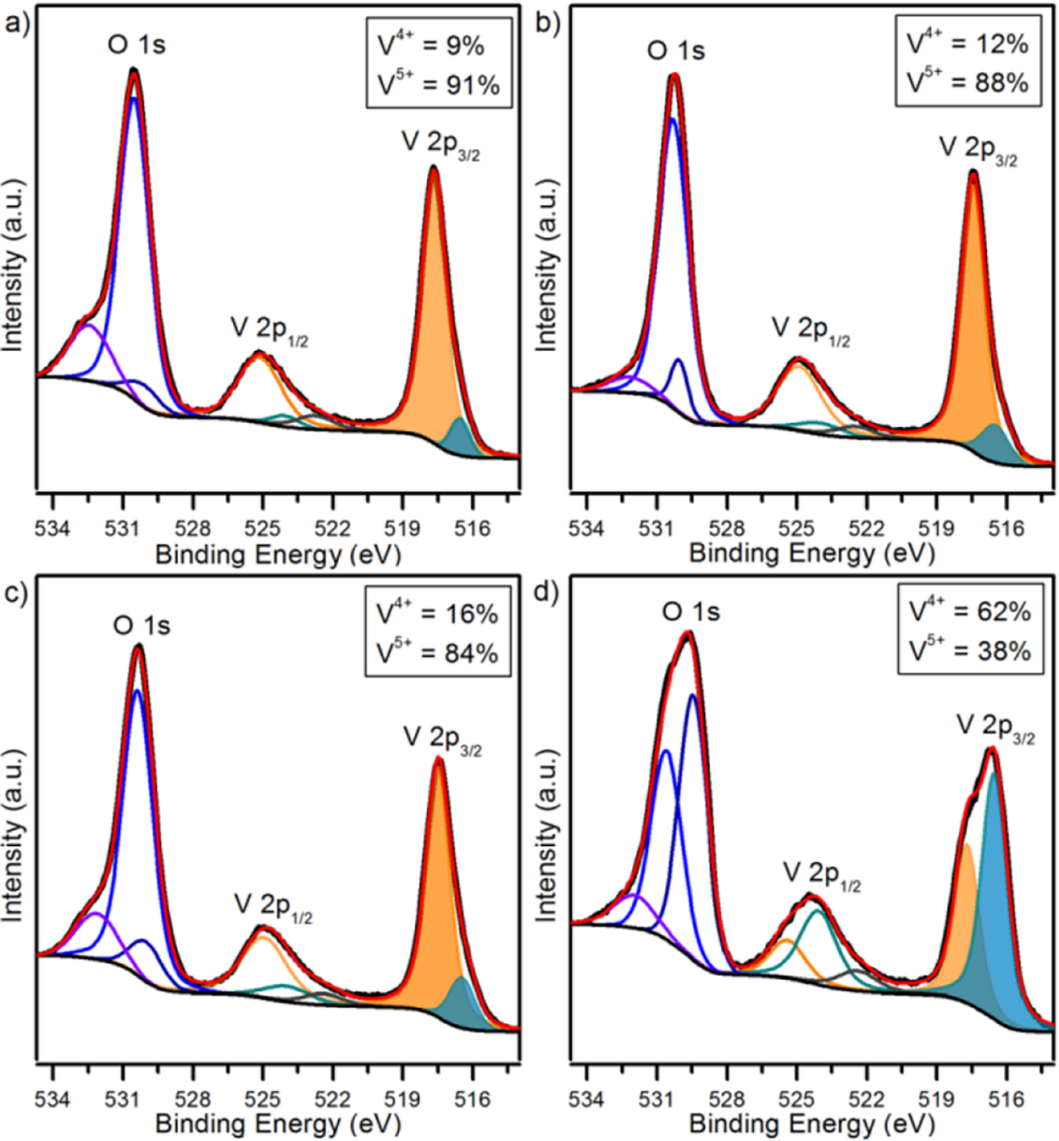
The XPS spectra of the O 1s and V 2p region of (a) KVO-20, (b) KVO-40, (c) KVO-60, and (d) KVO-80, respectively.

X-ray photoelectron spectroscopy (XPS) was used to assess the chemical composition and the charge state of vanadium ions in the studied samples. The high-resolution XPS spectra recorded in O 1s and V 2p binding energy regions (Figure 2a–d) show a complex shape of not less than three peaks located around 530 eV attributed to O 1s, as well as 517 and 524 eV attributed to the V 2p_3/2_ and V 2p_1/2_ peak doublet.^59^ The V 2p peaks for all samples show an asymmetric shape with a very weak shoulder line shifted toward negative binding energies versus the primary component. Based on these findings, two different V components should be used for spectral deconvolution. The V 2p_3/2_ peaks at 516.5 and 517.5 eV correspond to V^4+^ and V^5+^, respectively.^59^ Finally, the O 1s satellite at 522 eV was also considered in the deconvolution model based on previous findings.^60^ The XPS analysis allows one to estimate the relative share of vanadium V^4+^ to be on a similar level (9–16%) for the samples KVO-20, KVO-40, and KVO-60, whereas for the sample KVO-80, the vanadium V^4+^ valence state is dominant and equals 62%. The revealed significant difference in V^4+^ affects photocatalytic properties, originating from a narrow band gap of V^4+^ species facilitating charge carrier separation. The O 1s core-level spectra could be fitted by three components. The two most notable peaks at 530.1 and 530.5 eV are attributed to the O 1s orbitals of the O–V^4+^ and O–V^5+^ bonds, respectively, whose ratios are in good agreement with the V^5+^/V^4+^ ratios estimated from the deconvolution of V 2p_3/2_. The third peak centered at 532.5 eV, observable for all samples, is ascribed to adsorbed OH^–^ ions on the surface.^61^

The above XPS analysis confirms the mixed-valence character of the obtained samples and the existence of V(IV) and V(V) in their structure. However, it should be borne in mind that XPS is a surface-sensitive technique and that the obtained results indicate a high content of V^4+^ on the surface (which is important in the view of catalytic action) and not in the bulk. Thus, to further investigate the valence of vanadium in the materials, static magnetic susceptibility was measured for samples obtained at the lowest (KVO-20) and highest temperature (KVO-80).

**Figure 3:**
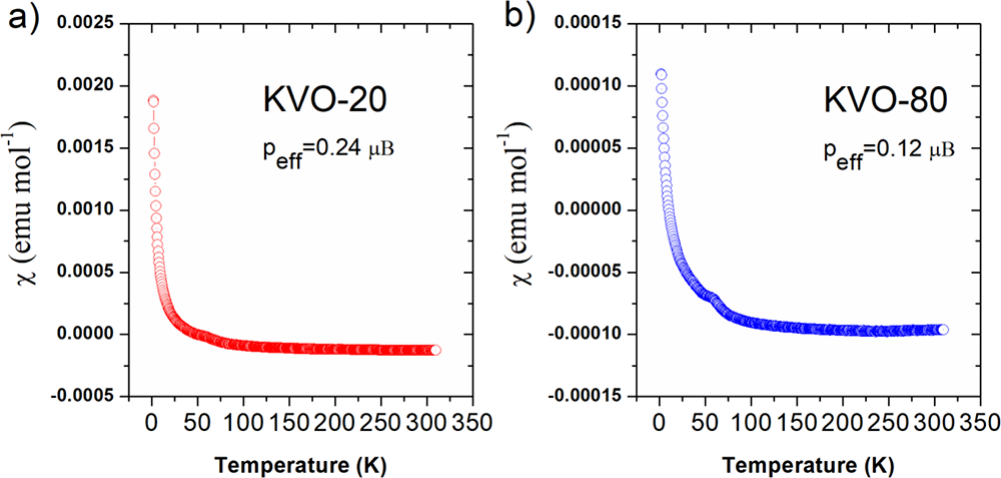
The temperature dependence of the magnetic susceptibility for (a) KVO-20 and (b) KVO-80 samples.

The temperature dependence of the magnetic susceptibility for KVO-20 and KVO-80 measured in an applied field of 3 T is shown in Figure 3. It can be clearly seen that for both samples above about 50 K, χ(*T*) is weakly temperature-dependent and its magnitude is small. At low temperatures, the pronounced tail is observed, which can be attributed to the presence of a small amount of uncompensated V^4+^ ions.^62^ To determine the effective magnetic moment (*p*_eff_), the experimental data were fitted by the modified Curie–Weiss law, χ = χ_0_ + *C*/(*T* – θ_P_), where χ_0_, *C*, and θ_P_ are the temperature-independent susceptibility, the Curie constant, and the paramagnetic Curie temperature, respectively. The fit gave *C* = 0.00712(3) emuK for KVO-20 and *C* = 0.00178(2) emuK for KVO-80. Assuming that the magnetic moment originates from V^4+^ only, the effective magnetic moment per V can be obtained using the relation *p*_eff_ = (3*Ck*_B_/μ_B_^2^*N*_A_)^1/2^, where *k*_B_ is the Boltzmann constant, μ_B_ is the Bohr magneton, and *N*_A_ is Avogadro’s number. The resulting effective magnetic moment *p*_eff_ is 0.24 and 0.12 μ_B_/V for KVO-20 and KVO-80, respectively. The calculated effective magnetic moments are much smaller than the expected value of *p*_eff_ = 1.73 μ_B_ for the free V^4+^ ion. Thus, obtained results suggest that the samples consist of a vast majority of nonmagnetic V(V) species and only a small fraction of V(IV) located on the sample surface or between crystalline grains, and in case of bulk samples, the V^4+^/V^5+^ ratio is smaller than that on the surface.

**Figure 4:**
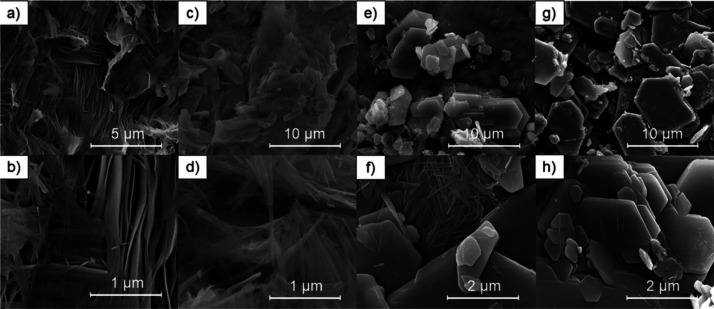
SEM images of samples (a, b) KVO-20, (c, d) KVO-40, (e, f) KVO-60, and (g, h) KVO-80.

**Figure 5:**
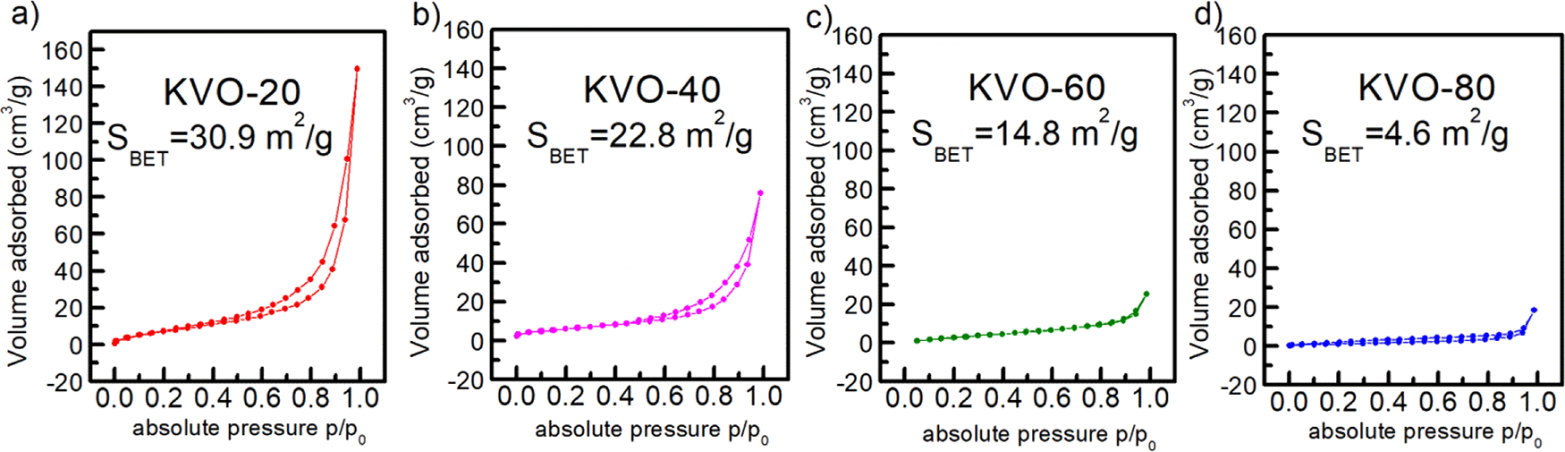
N_2_ adsorption–desorption isotherm and calculated surface area for (a) KVO-20, (b) KVO-40, (c) KVO-60, and (d) KVO-80.

The morphologies of the samples undergo significant temperature evolution. The sample KVO-20 (single-phase K_2_V_6_O_16_·0.65H_2_O according to XRD, FTIR, and TG results) consists of belt-like structures with a width between 50 and 200 nm, a thickness of 10–40 nm, and a length of a few micrometers (Figure 4a,b). With the increase of the synthesis temperature, the K_2_V_6_O_16_·0.65H_2_O nanobelts become thinner, and their cross-sections decrease. For samples KVO-40 and KVO-60, the width of K_2_V_6_O_16_·0.65H_2_O nanostructures is between 30 and 50, 20 and 40, and 10 and 20 nm, respectively, while the thickness of nanobelts is below 10 nm. Moreover, the K_2_V_6_O_16_·0.65H_2_O nanobelts’ length decreases from a few micrometers (sample KVO-40) to 100–250 nm (sample KVO-60). The crystals of the secondary phase (KV_3_O_8_) exhibit a plate-like morphology, and for sample KVO-40 (Figure 4c,d), the width of plates is between 0.5 and 2.5 μm, the length is between 0.5 and 4 μm, and the thickness is about 50 nm. With increasing synthesis temperature (sample KVO-60 and KVO-80), the length of KV_3_O_8_ crystal rises up to 0.5–7 and 1–10 μm, respectively, and the width of crystals equals ca. 150 nm. The thickness of KV_3_O_8_ crystals in the single-phase sample KVO-80 (Figure 4g,h) is more uniform and equals 150 nm, whereas for the multiphase sample KVO-60 (Figure 4e,f), it is in the range between 50 and 350 nm. Moreover, the surface areas of samples were determined through N_2_ adsorption and BET analysis (Figure 5). As expected, the highest surface area (30.9 m^2^/g) has been noted for KVO-20 with a nanostructural morphology and the lowest (4.6 m^2^/g) for KVO-80 with microplatelets.

**Figure 6:**
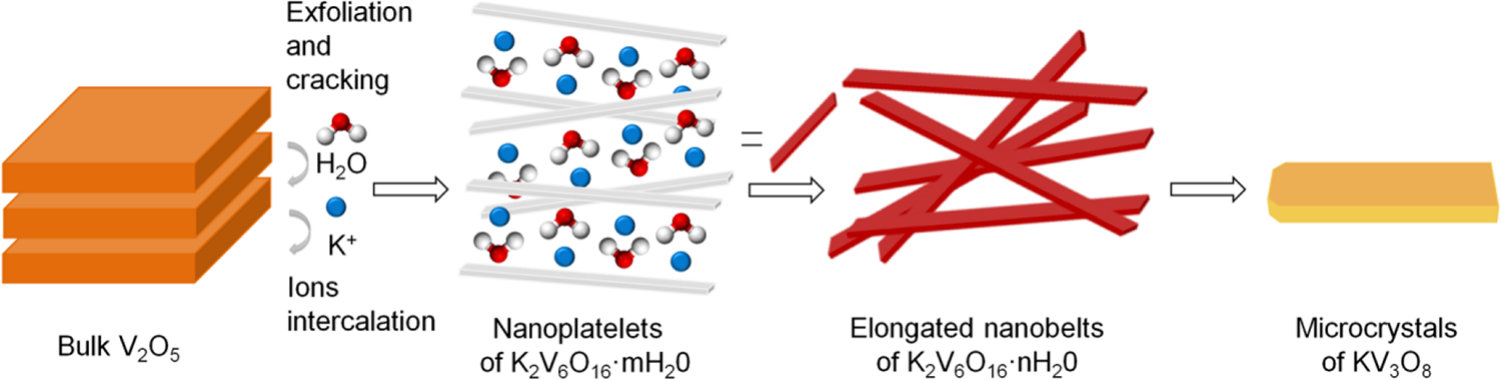
Schematic illustration of the evolution of potassium vanadate morphology.

The schematic illustration of the evolution of potassium vanadate morphology is presented in Figure 6. The synthesis reaction is based on the intercalation of the solvent molecule into layer spacing and its further exchange with alkali metal ions. The reaction scheme of KVO by the LPE-IonEx synthesis method is similar to the ammonium metavanadate synthesis reported previously.^63^ The solid vanadium oxide underwent delamination by the insertion of water molecules into the interlayer space. The H_2_O molecules are partially trapped during potassium ion intercalation, and hydrated potassium vanadate (K_2_V_6_O_16_·*m*H_2_O) is formed. During the synthesis at room temperature, the water stoichiometry is stabilized and elongated nanobelts (K_2_V_6_O_16_·*n*H_2_O) are obtained. The KV_3_O_8_ microcrystals are formed via the dehydration and recrystallization of K_2_V_6_O_16_·*m*H_2_O at higher synthesis temperatures because the exchange between solvent molecule and potassium ion is more efficient, and a nonhydrated phase is obtained: KV_3_O_8_. Throughout the dehydration process, the *c* axis of the crystal unit cell is decreased by almost half. During this process, the creation of V^4+^ species is probable. Therefore, the obtained KV_3_O_8_ phase possesses a rich vanadium-defective structure.

### Photocatalytic Properties

**Figure 7:**
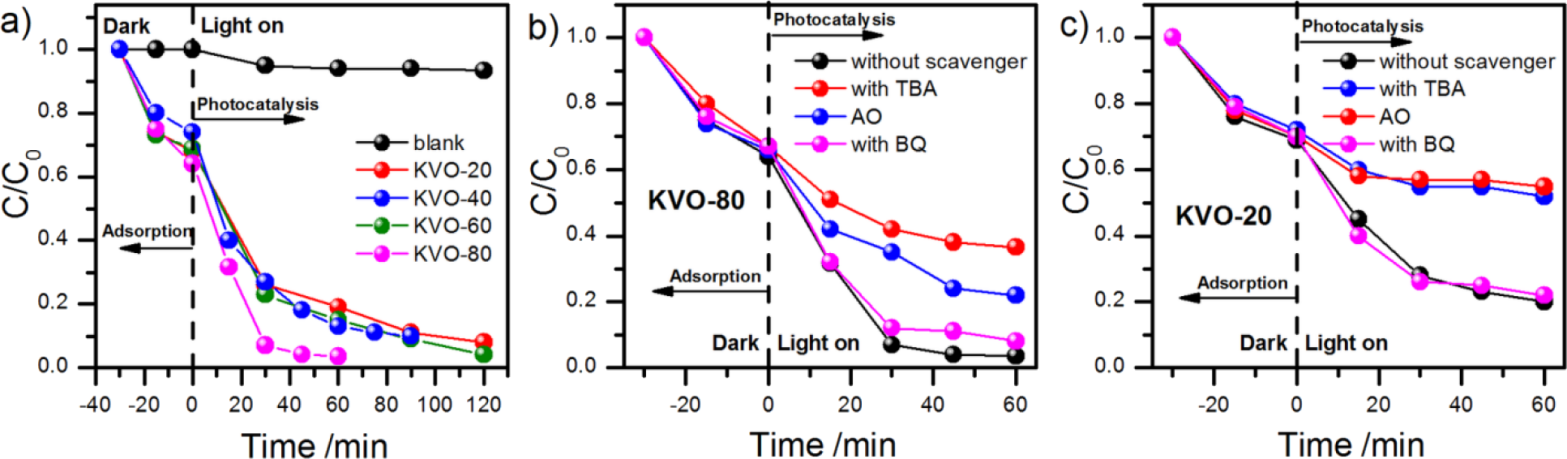
(a) The *C*/*C*_0_ vs *t* plot representing the photocatalytic degradation of MB for all tested samples. (b, c) The effect of scavengers on the MB degradation rate for single-phase samples KVO-80 (KV_3_O_8_) and KVO-20 (K_2_V_6_O_16_·0.65H_2_O), respectively.

The photocatalytic performance of investigated powders is presented as a *C* to *C*_0_ ratio in Figure 7a. Additionally, the effect of the photolysis of MB is added for comparison (blank). Before the irradiation, methylene blue was adsorbed on the surface of the tested powders at a similar, considerable level of 30%. Adsorption is an essential process in photocatalysis, which enriches the pollutants around the catalyst surface, and often, strong adsorption results in increased photocatalytic performance.^64^ All samples acted as efficient photocatalysts for dye decomposition; however, the sample obtained at 80 °C exhibited the highest photoactivity. After about 30 min of exposure, over 90% of MB was degraded. In the case of the rest of the samples (obtained at 20, 40, and 60 °C), the observed photoactivity was similar and the time required for MB degradation was longer (approximately 90% of degradation after 90 min). To the best of our knowledge, there is only one report^65^ devoted to the potassium vanadate compounds that presents using of hydrated vanadates (K_2_V_6_O_16_·1.5H_2_O/2.7H_2_O) for the photodegradation of methyl orange. In comparison to the mentioned work, samples obtained by our method exhibit better photocatalytic activity. Furthermore, the calculated band gap for K_2_V_6_O_16_·*n*H_2_O is narrower in the case of the sample reported by us. Moreover, herein, for the first time, we present nonhydrated KV_3_O_8_ as a new efficient photocatalyst. This implies that the photocatalytic efficiency of our samples results from the specific structural properties, which were provided by the proposed innovative LPE-IonEx method. Often, the developed surface also has a beneficial effect for photocatalytic efficiency. It is reported that a high surface area inhibits the electron–hole recombination and allows the adsorption of more MB molecules.^66−68^ Nonetheless, for investigated samples, the reverse tendency was observed. The sample with the lowest surface area (according to the BET method) exhibited the best photocatalytic performance, while for the sample with the highest surface area, the degradation process was the least effective. This confirms that, in the case of the synthesized samples, their photocatalytic activity is not dependent on the surface area. It is supposed that the presence of V^4+^ species plays a pivotal role here. Surface defect engineering has gained a lot of attention in the development of efficient photocatalysts. The existence of defect sites in vanadates has been reported to be crucial in enhancing photocatalytic performance in water splitting and degradation of pollutants.^69−71^ Zhang et al. have shown enhanced photoelectrochemical performance of V^4+^ self-doped m-BiVO_4_. Compared to the pure BiVO_4_, V^4+^ doped samples possess enhanced photocurrent density, smaller charge transfer resistance, longer electron lifetime, and improved separation of photogenerated electrons and holes.^72^ Yu and co-workers presented CaV_2_O_6_ nanorods that exhibited improved photocatalytic activity in MB degradation due to the coexistence of V^4+^/V^5+^ in the lattice. CaV_2_O_6_ shows an indirect allowed electronic transition with a band gap energy of 2.56 eV and hydroxyl radicals as the major active species.^73^ Saputera et al. obtained BiVO_4_ with a different vanadium vacancy content by changing the calcination temperature after sol–gel synthesis. Obtained samples were then evaluated by the degradation of palm oil mill effluent waste under UV–visible light. Results revealed that the higher the V^4+^ content was, the better were the degradation rates and reaction rate constant. It was also observed that the increase in V^4+^ content resulted in a slight increase of band gap energy (2.44 to 2.50 eV).^74^

To investigate the kinetics of the process, the ln(*C*/*C*_0_) vs *t* plot was prepared (see Supporting Information Figure S2). The most probable is the pseudo-first-order kinetics according to the Langmuir–Hinshelwood model;^75^ however, the mechanism of the photocatalytic degradation can be more complex. Thus, the additional measurements for single-phase samples K_2_V_6_O_16_·0.65H_2_O (KVO-20) and KV_3_O_8_ (KVO-80) in the presence of appropriate scavengers were performed (see Figure 7b,c). *tert*-Butyl alcohol (TBA), benzoquinone (BQ), and ammonium oxalate (AO) act as a hydroxyl radical, superoxide radical, and hole scavenger, respectively.^76^ The decrease of the decomposition efficiency in a solution containing a scavenger suggests the role of the relevant species in the mechanism of the degradation mechanism. Thus, in both cases, the role of superoxide radicals is negligible. On the other hand, degradation efficiency in the presence of a hydroxyl radical scavenger is significantly diminished in comparison with the control measurement. It is indirect proof that OH· is formed during the illumination of the K_2_V_6_O_16_·0.65H_2_O (KVO-20) and KV_3_O_8_ (KVO-80) aqueous suspensions. Noteworthily, the direct oxidation of MB by photoexcited holes from the valence band of the photocatalyst also takes part in the degradation process. According to the results presented in Figure 7b, it can be concluded that approximately equal amounts of MB are decomposed by OH radicals and holes in the case of K_2_V_6_O_16_·0.65H_2_O. The role of direct oxidation in the case of KV_3_O_8_ is smaller but still significant (Figure 7c).

### Optical Properties of K_2_V_6_O_16_·0.65H_2_O Nanobelts and KV_3_O_8_ Microplatelets

**Figure 8:**
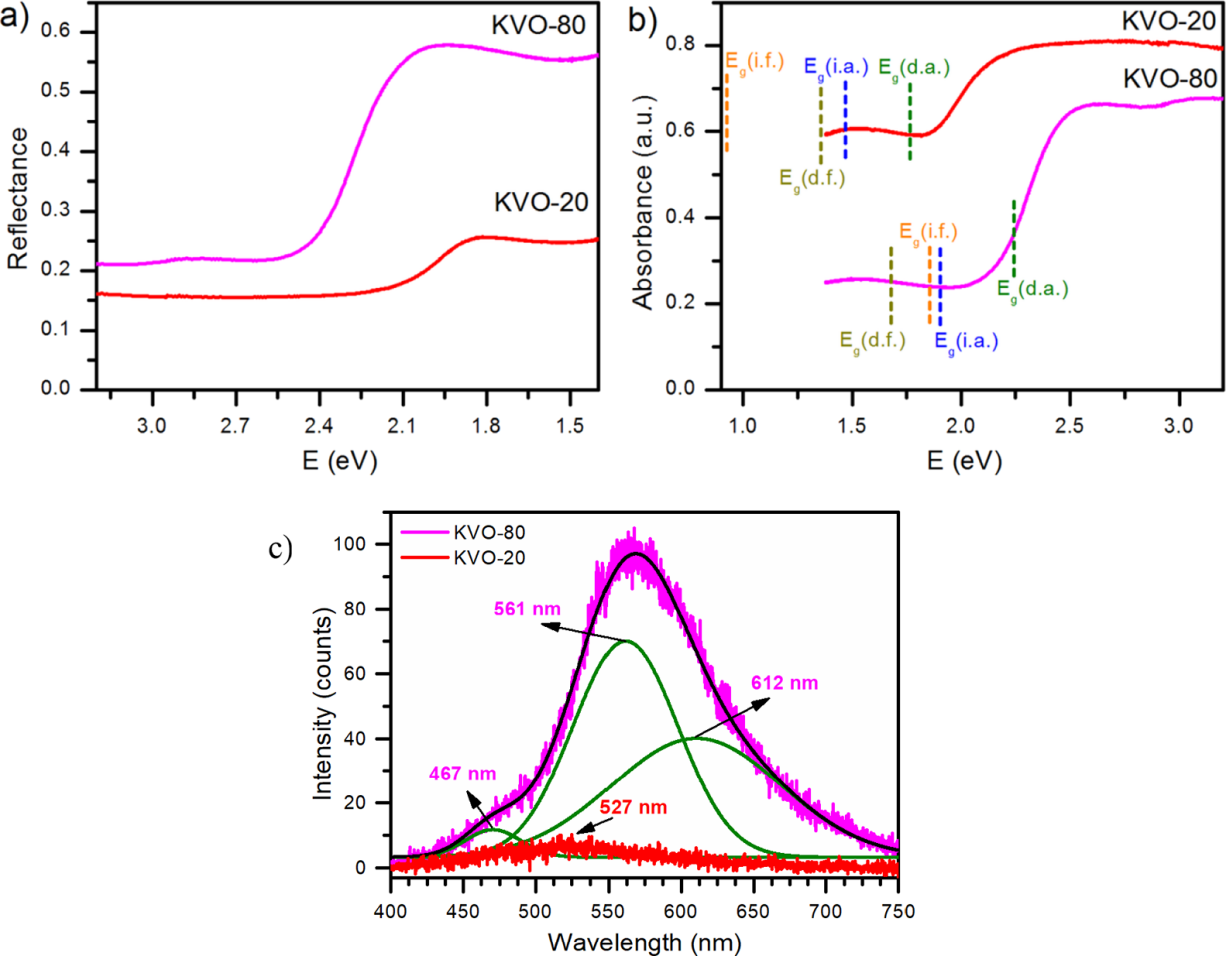
(a) Reflectance (measured) and (b) absorbance (calculated) spectra of KV_3_O_8_ microplatelet (KVO-80) and K_2_V_6_O_16_·0.65H_2_O nanobelt (KVO-20) samples. (c) Photoluminescence spectra of KV_3_O_8_ (KVO-80) and K_2_V_6_O_16_·0.65H_2_O (KVO-20).

The optical behavior of single-phase samples KV_3_O_8_ microplatelets and K_2_V_6_O_16_·0.65H_2_O nanobelts, which were, respectively, the most and the least photoactive material, was further examined using UV–vis spectroscopy in the reflectance mode. The spectra of both samples are presented in Figure 8a. As can be seen, synthesized materials absorb a significant part of the light in the visible range. This feature is beneficial for the materials used in photocatalysis. Reflectance edges seen on both spectra are related to the energy band gap transition, so the obtained results allowed the energy band gap to be estimated. There is no information about the type of electron transition; thus, all possibilities were taken into account, as presented in Figure S3. The absorbance spectra of both powders with marked band gaps estimated from (*f*(KM)*·h*ν)^*n*^ vs *h*ν plots are presented in Figure 8b. Since the energy band gap corresponds to the absorbed photons with the lowest energy, the direct energy band gap (d.a.) in the case of K_2_V_6_O_16_·0.65H_2_O nanobelts is the most probable. On the other hand, the estimated value of the direct band gap for KV_3_O_8_ microplatelets corresponds to a wavelength that is already within the absorption edge. Thus, it is very likely that the optical band gap of the KV_3_O_8_ material is related to the allowed indirect transition (i.d.); nevertheless, the direct band gap can be taken into account as well. Thus, the determined direct energy band gaps for K_2_V_6_O_16_·0.65H_2_O and KV_3_O_8_ are 1.80 and 2.23 eV (and the indirect energy band gap for KV_3_O_8_ is equal to 1.91 eV), respectively. These values are in accordance with the observed colors: dark red for the hydrated phase and orange for the nonhydrated phase. The differences in estimated energy band gaps suggest that KVO-20 could be a better photocatalyst due to the wider range of electromagnetic radiation that can be absorbed and converted. As shown in Figure 7, it is not the case. In the case of KV_3_O_8_ and K_2_V_6_O_16_·0.65H_2_O, direct comparison is misleading because they are two different compounds with different crystal structure and stoichiometry (Figure 1), surface composition (Figure 2), morphology (Figure 4), and surface area (Figure 5). It can be concluded that all the above-mentioned parameters have a greater impact on photoactivity than UV–vis absorption ability.

Additionally, the photoluminescence spectra have been recorded using UV excitation. Results are presented in Figure 8c. Generally, the emission spectrum of KVO-80 is characterized by a much higher intensity of photoluminescence in comparison with KVO-20. It could be simply related to the better crystallinity of the sample prepared at a higher temperature;^77^ however, the shape of the emission bands is also different. Often, photocatalysts are examined with PL to compare emission intensity. In the case of photoactive materials, a higher PL intensity means a higher rate of recombination processes and therefore poorer photocatalytic performance. Thus, here PL spectra suggest that better photocatalytic activity should be observed for the KVO-20 sample (which is not true). However, such direct comparison can be done for samples with similar characteristics (composition, morphology, surface area, etc.) because PL intensity is affected by many parameters.^78^

The PL intensity of the KVO-20 spectrum is very low, which makes the analysis very difficult to perform. The maximum can be found at about 527 nm (2.35 eV). Taking into account the absorbance spectrum, the recorded emission is not related with conduction to valence band extinction. In the case of the KVO-80 sample, the spectrum can be deconvoluted to 3 Gaussian peaks with the maximum at 467 nm (2.66 eV), 561 nm (2.21 eV), and 612 nm (2.03 eV). The PL emission at energies lower than the band gap (2.03 eV) is related to the presence of states within the energy band gap. Here, it can be associated with the presence of oxygen vacancies and V(IV) centers seen on XPS. A similar phenomenon was observed for partially reduced metal oxides (TiO_2_, SnO_2_, and In_2_O_3_), while for fully oxidized materials, visible light photoluminescence was diminished.^79^ The PL bands at energies lower than the energy band gap were also reported for V_2_O_5_ and were described as an effect of the oxygen vacancies’ presence.^80^ The second band at the PL spectrum of KVO-80 has an energy (2.21 eV, 561 nm) close to the band gap; thus, it probably originates from the direct band edge transition. The low-intensity band at 467 nm that can be seen in Figure 8c and a similar one were already reported for V_2_O_5_ nanostructures. It was described as the recombination of UV excited electrons from a higher level in the V 3d orbital (in the conduction band) to the ground level.^81^

### Energy Band Position of KV_3_O_8_ (KVO-80)

**Figure 9:**
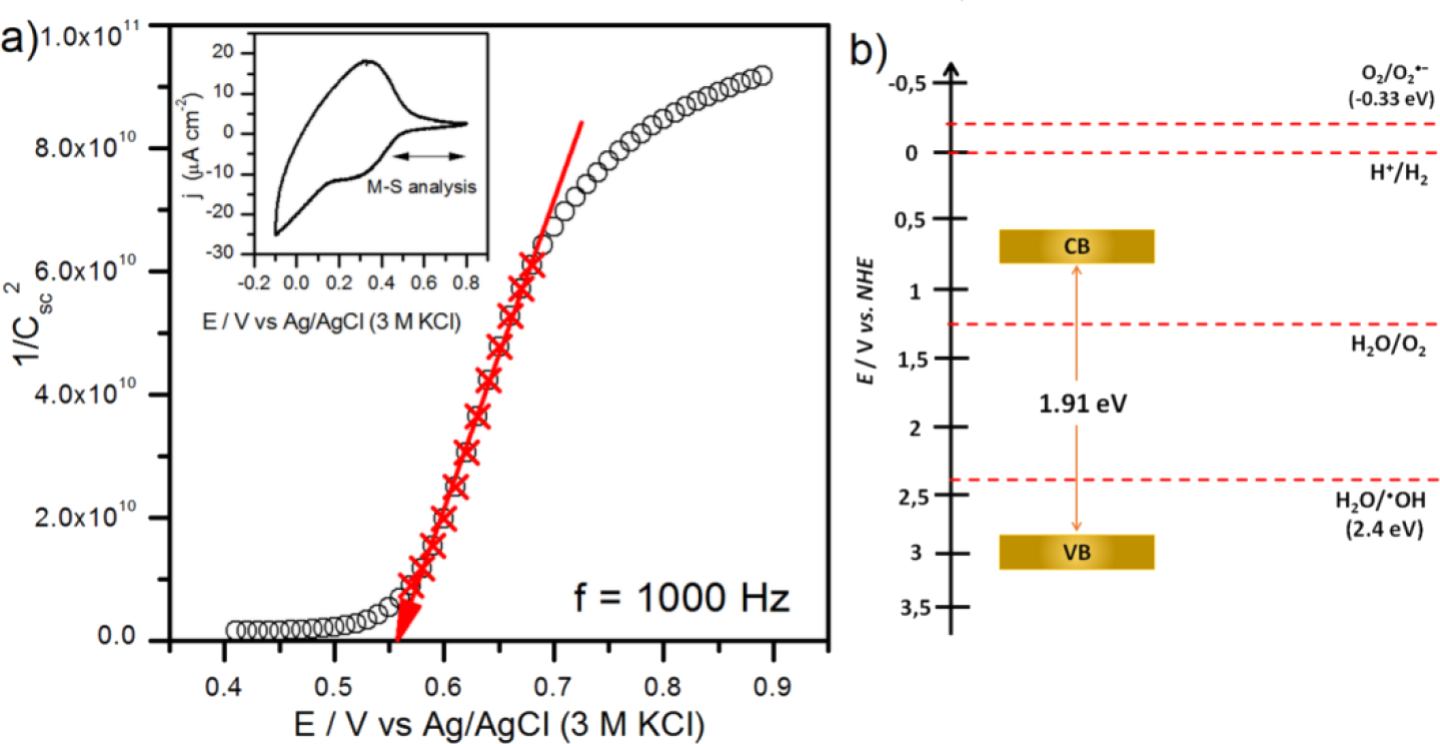
(a) The Mott–Schottky analysis performed for KV_3_O_8_ (KVO-80). Inset: cyclic voltammetry curve recorded with 50 mV/s. (b) The energy band position diagram of the sample KV_3_O_8_ (KVO-80).

Electrochemical characterization of the sample allowed the estimation of the energy band position for the stable nonhydrated KV_3_O_8_ phase. The procedure of sample thin film preparation did not affect its crystal structure and the charge state of vanadium (see Supporting Information Figures S4 and S5). The flat band potential (*E*_fb_) was determined using the Mott–Schottky (MS) analysis. To estimate the space charge region capacitance (*C*_sc_), the impedance spectra were measured at dark conditions as a function of applied potential. The range of potential for MS analysis was chosen on the basis of the cyclic voltammetry (CV) study presented in the Figure 9a inset. The peaks observed at the CV curve are probably related to the V center oxidation/reduction with the simultaneous K^+^ intercalation/deintercalation process. The impedance spectra were measured between 0.4 and 0.9 V vs Ag/AgCl (3 M KCl), but the linear behavior of 1/*C*_sc_^2^ vs *E* was found between 0.58 and 0.69 V (see Figure 8a). The positive slope of the Mott–Schottky plot confirmed that the obtained nonhydrated KV_3_O_8_ phase is an n-type semiconductor and *E*_fb_ equals to about 0.56 V vs Ag/AgCl (3 M KCl) (0.765 V vs NHE). The energy band diagram was prepared assuming that *E*_fb_ lies just below the conduction band and the UV–vis determined indirect energy band gap of KV_3_O_8_ is 1.91 eV. As presented in Figure 8b, the valence band (VB) location allows photoexcited holes to react with adsorbed water and form hydroxyl radicals. On the other hand, the potential of the photoexcited electrons from the conduction band (CB) is not enough to form superoxide radicals. These conclusions are consistent with the efficiency of the photocatalysts.

According to the above-described results, the possible mechanism of photocatalytic performance of KV_3_O_8_ has been proposed. The major reaction steps responsible for the photocatalytic activity could be expressed as follows:

(1)
(2)
(3)
(4)
(5)

The UPLC–QTOF-MS/MS was further applied to identify the main MB degradation products for samples obtained after photocatalytic degradation by KVO-80 (KV_3_O_8_) and KVO-20 (K_2_V_6_O_16_·0.65H_2_O) used as a catalyst. The total ion current chromatograms (TICs, Supporting Information) are presented in Figure S6. On the basis of the received data after using KVO-80 as a catalyst, besides the nondecomposed MB, two additional degradation compounds were identified. The observed peaks on the chromatogram at 2.576 and 8.122 min correspond probably to 4-nitroaniline [C_6_H_6_N_2_O_2_ + H]^+^ and *m*/*z* 139.0512, and phenol [C_6_H_6_O + H]^+^ and *m*/*z* 95.0494, respectively (see Figure S7, Supporting Information), while the peak observed at 4.591 min is attributed to MB. The above photodegradation process generates about 8.34% of nondecomposed MB, 0.12% of aniline, and 0.45% of phenol. The possible degradation pathway where the aniline occurred as a product was described elsewhere.^82^

In the case of analysis of MB degradation products using KVO-20 as the photocatalyst, the obtained results allowed for the identification of at least four additional compounds besides MB residues (4.586 min, see Figure S8). The observed peaks on the chromatogram at 2.604 and 8.116 min in the resulting products, as in the case of KVO-80 catalyst, can be assigned to 4-nitroaniline [C_6_H_6_N_2_O_2_ + H]^+^ and *m*/*z* 139.0508, and phenol [C_6_H_6_O + H]^+^ and *m*/*z* 95.0494, respectively. Analysis of the sample after the use of KVO-20 as a catalyst also identifies two additional compounds. On the chromatogram at 3.12 min, the [C_7_H_10_N_2_O_4_S + H]^+^ and *m*/*z* 219.0444 was identified, corresponding to 2-amino-5-(methylamino)-hydroxybenzenesulfonic acid, which was previously described in the photocatalytic MB degradation pathway,^83^ which leads to H_2_O and CO_2_. In addition, it is worth mentioning that on the chromatogram peak presence at 5.219 min reveal to benzenesulfonic acid [C_6_H_6_O_3_S + H]^+^ and *m*/*z* 159.0063, which also occur in the photocatalytic MB degradation pathway as previously described.^84^ Comparing the efficiency of MB degradation through the use of the KVO-20 catalyst with the KVO-80 catalyst, the efficiency of the KVO-20 catalyst is significantly lower. The photodegradation process catalyzed by KVO-20 generates about 16.78% of nondecomposed MB, 36.27% of benzenesulfonic acid, and comparable amounts (0.40%) of phenol and the amino-5-(methylamino)-hydroxybenzenesulfonic acid, which is formed in approximately 0.46%. On the other hand, a negligible 0.07% amount of 4-nitroaniline is formed when the KVO-20 catalyst was applied, which indicates the efficiency and difference of the photodegradation mechanisms of MB using the described catalysts.

## Conclusions

In this study, K_2_V_6_O_16_·0.65H_2_O nanobelts and KV_3_O_8_ microplatelets were synthesized using the facile LPE-IonEx method and investigated as catalysts for organic dye degradation. The obtained samples were characterized using XRD, FTIR, XPS,PPMS, UV–vis DRS, PL, TGA with MS, SEM, and N_2_ adsorption isotherms. Controlling the reaction temperature (20–80 °C) resulted in various phase compositions, morphologies, and surface areas. Moreover, different synthesis temperatures provided varied concentrations of V^4+^ (from 9% up to such a high content as 62%). The photocatalytic degradation of methylene blue, under simulated solar light illumination, was examined to evaluate the photocatalytic performance of prepared samples. All samples acted as efficient photocatalysts, resulting in approximately 90% degradation of the dye within the first 30–90 min. The highest activity was observed for the sample obtained at 80 °C. According to the scavenger test, OH radicals and holes are the main active species in the case of K_2_V_6_O_16_·0.65H_2_O. The role of direct oxidation in the case of KV_3_O_8_ is smaller but still significant. The results indicate that potassium vanadates are potential candidates for light-driven photocatalysts. The observed excellent photocatalytic performances result from the specific structural properties, which were provided by the proposed innovative LPE-IonEx method. We suggest that it can be attributed to the high content of V^4+^ species in the samples, which traps electrons and facilitates charge separation.

## Supporting Information Available

TG and DTG curves with ion current curves of the KVO-20 sample; DSC curves of the KVO-20 and KVO-80 sample; FTIR, XRD, and SEM image of sample KVO-20 after heat treatment; XRD of as-prepared sample KVO-80 and XRD and XPS of KVO-80 deposited onto degreased FTO (KVO-80/FTO); results of MB photodegradation presented as the ln(*C*/*C*_0_) vs time plots; and UPLC–QTOF-MS/MS (chromatograms and mass spectra) of MB degradation products for samples obtained after photocatalytic degradation by KVO-80 and KVO-20 (PDF)
