# Supplementary material for: Insight into Potassium Vanadates as Visible-Light-Driven Photocatalysts: Synthesis of V(IV)-Rich Nano/Microstructures for the Photodegradation of Methylene Blue
Source: Inorg Chem. 2022 Jun 10;61(25):9433–44. doi: 10.1021/acs.inorgchem.2c00136 (PMC9241143; doi:10.1021/acs.inorgchem.2c00136)
Supplement: Supplementary file 1 — ic2c00136_si_001.pdf [file ic2c00136_si_001.pdf]

Insight into potassium vanadates as visible-light-driven photocatalysts: synthesis of V(IV)-rich nano/microstructures for photodegradation of methylene blue

Małgorzata Nadolska[a], Mariusz Szkoda[b], Konrad Trzciński[b], Paweł Niedziałkowski[c], Jacek Ryl[a], Aleksandra Mielewczyk-Gryń[a], Karolina Górnicka[a], Marta Prześniak-Welenc[a*]

[a]*Faculty of Applied Physics and Mathematics, Institute of Nanotechnology and Materials Engineering, Gdansk University of Technology, Narutowicza 11/12, 80-233 Gdansk, Poland*

[b]*Faculty of Chemistry, Gdansk University of Technology, Narutowicza11/12, 80-233 Gdansk, Poland*

[c]*Faculty of Chemistry, University of Gdansk, Wita Stwosza 63, Gdansk, 80-308, Poland*

*Corresponding author; e-mail: marta.welenc@pg.edu.pl, tell: +48 583486606,

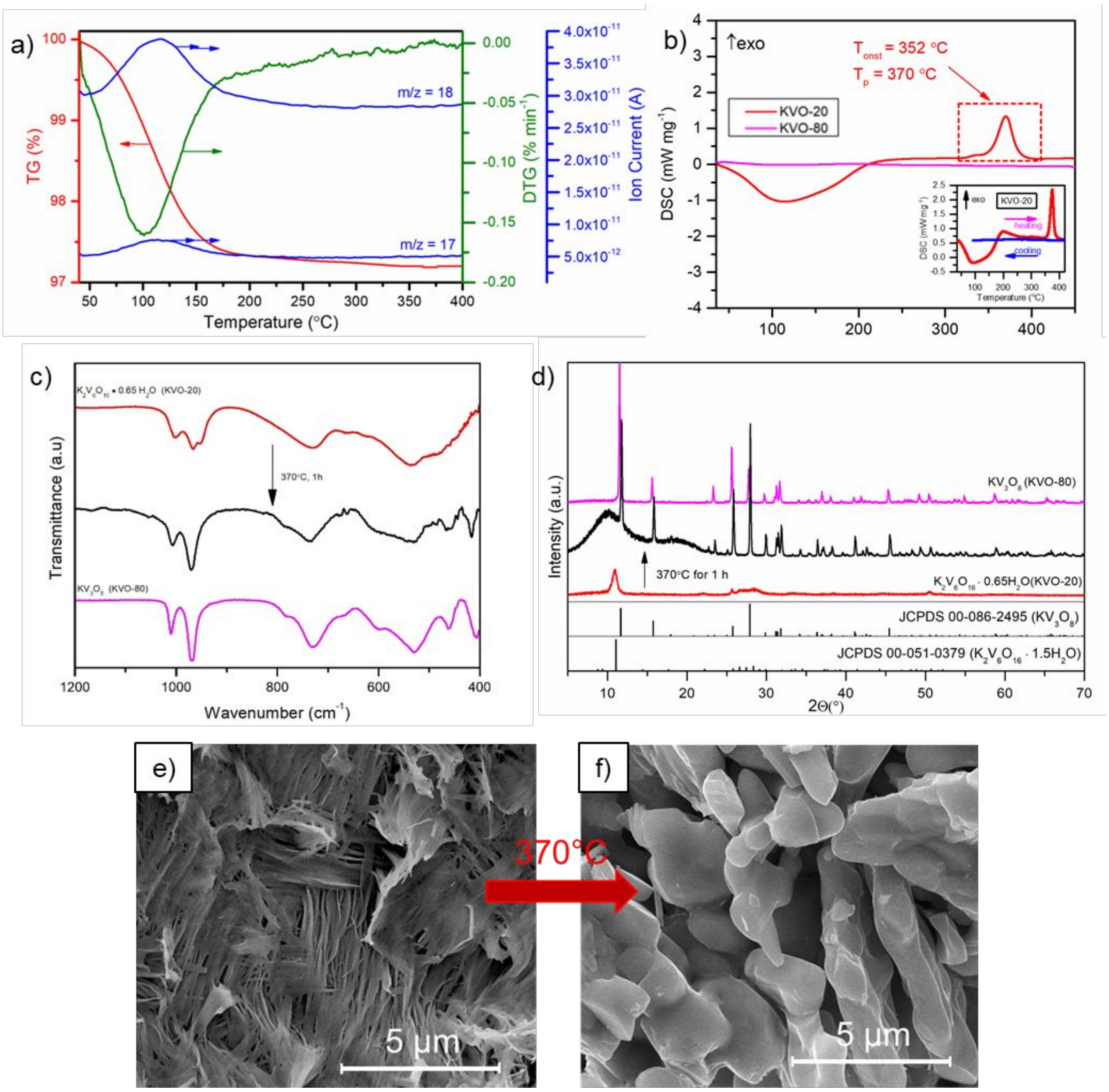

Figure S1.a) TG and DTG curves of the KVO-20 with Ion Current curves for m/z =18 and m/z = 17 b) DSC curves of the KVO-20 and KVO-80 sample, inset: DSC curves of the KVO-20 during heating and cooling rate, c) XRD patterns, d) FTIR spectra the KVO-20 after heat treatment at 370°C for 1h,e) and f) SEM images of sample KVO-20 before and after heat treatment at 370°C for 1h, respectively.

The asymmetric bell-shaped peak located between 35-250°C on DSC curve of KVO-20 (Fig. S1a) is caused by decomposition of $K_2V_6O_{16}\cdot0.65H_2O$ with the elimination of the physisorbed water and water of the crystallization. The asymmetric peak with a shoulder between 100-250°C on DTG curve (Fig. S1c) indicates stepwise water elimination. The peak located at 352°C (T_{onst}) is attributed to recrystallization of $K_2V_6O_{16}\cdot0.65H_2O$ to non-hydrated form KV_3O_8. To further confirm this assumption the KVO-20 (single phase, $K_2V_6O_{16}\cdot0.65H_2O$) sample was heated at 370 °C for one h and the SEM, FTIR and XRD analysis were performed. Both, XRD and FTIR confirm that after heating KVO-20 recrystallize to KV_3O_8 and FTIR spectrum and diffractogram are similar to that registered for KVO-80 (KV_3O_8).

Table S1. Summary of the IR bands position

		Band position (cm^{-1})	
	Vibrational mode	KVO-20	KVO-80
vandate	V=O streching vibrations	1001	1010
	V=O streching vibrations	966 952	968
	bridging V-O···K stretching	727	730
	V-O-V stretching	535	530/600
water	H-O-H bending	1615	1633
	O-H streching	3435	3475

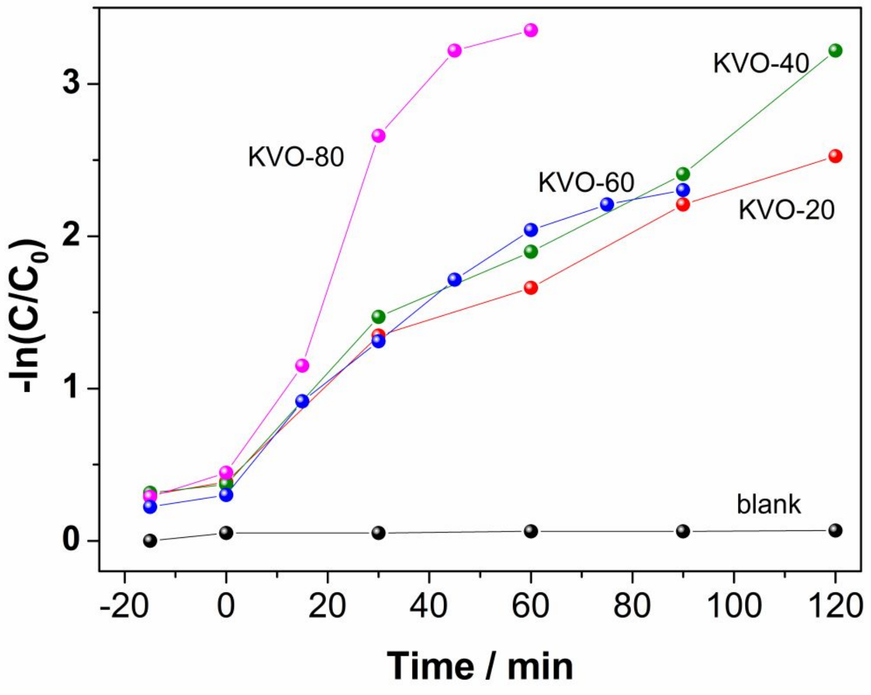

Figure S2.The results of MB photodegradation presented as the $\ln(c/c_0)$ vs time plots.

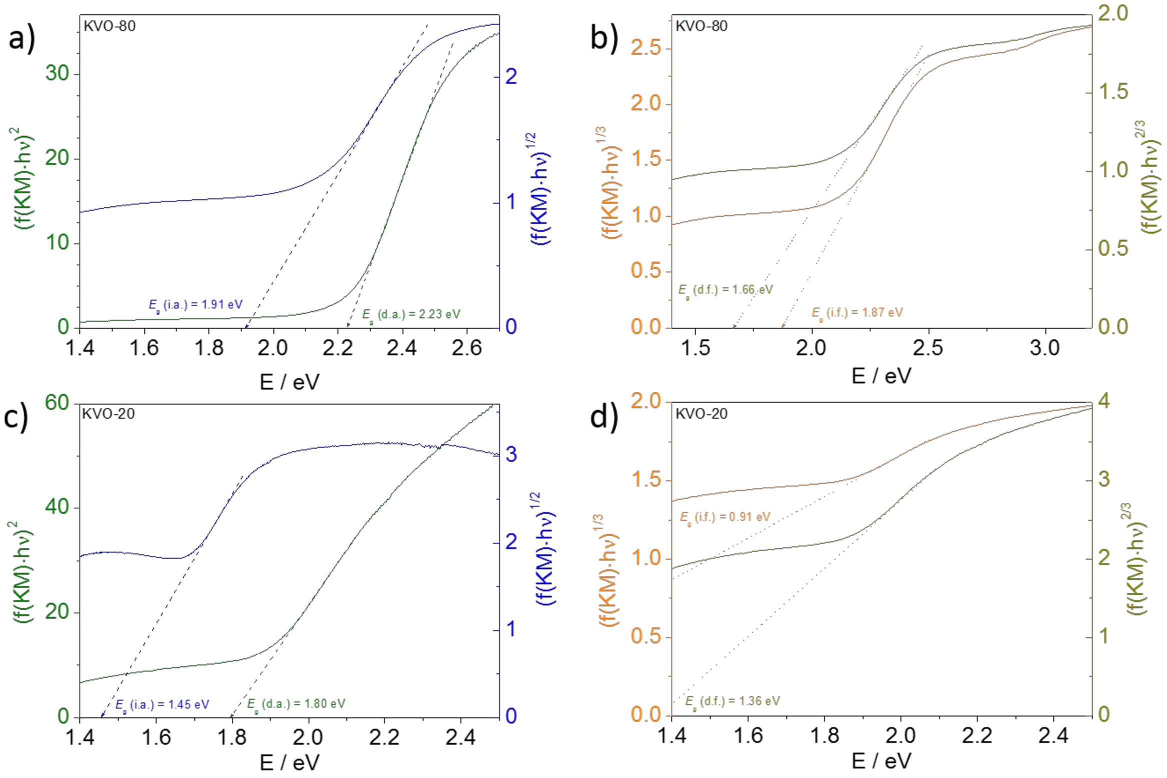

Figure S3. The $(f(KM)\cdot hv)^n$ vs. hv plots (n = 1/2 (a); 2 (b); 1/3 (c); and 2/3 (d)) resulting from the UV-Vis reflectance spectra of KVO-20 and KVO-80 powders.

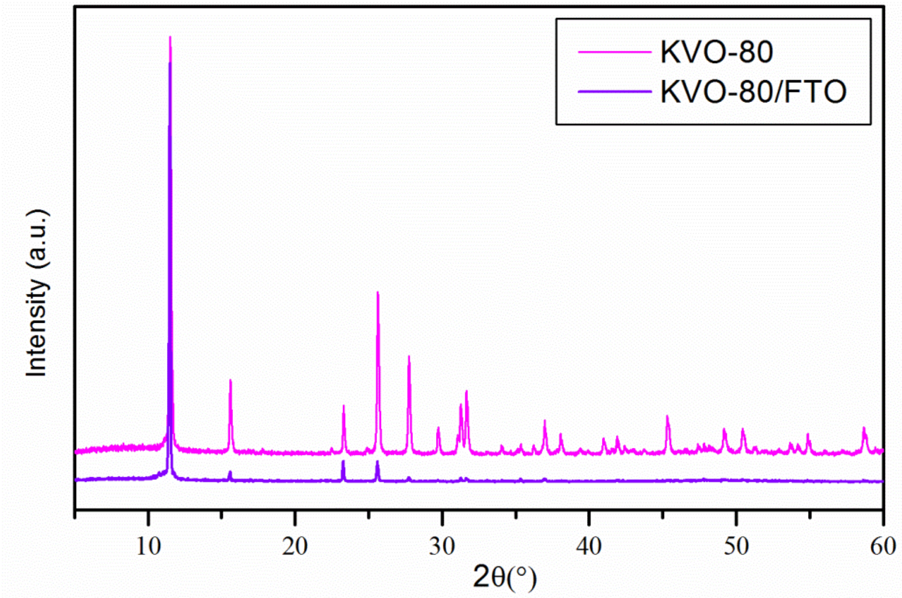

Figure S4.The X-ray diffractograms of as-prepared sample KVO-80 and KVO-80 deposited onto degreased FTO (KVO-80/FTO).

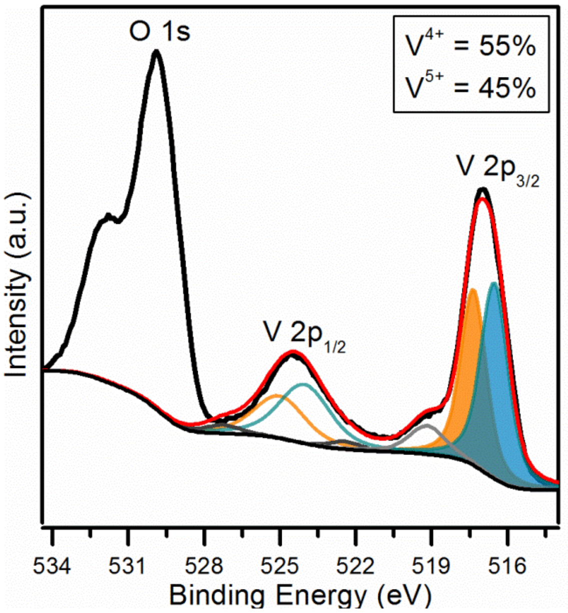

Figure S5.The XPS spectra of O1s and V2p region of KVO-80 sample deposited onto degreased FTO.

The two different components of V $2p_{3/2}$ are located around 516.5 eV and the 517.5 eV, and correspond to the V^{4+} and V^{5+}respectively [64]. These components are also present for V $2p_{1/2}$ peak, the two peaks centered at 525 eV (V^{5+})and 524 eV (V^{4+}). The peaks located around 522 and 519 eV were attributed to the O1s satellite [65]. Based on the area of the fitted curves, the relative atomic percentage of vanadium V^{+4} was estimated to 55%.

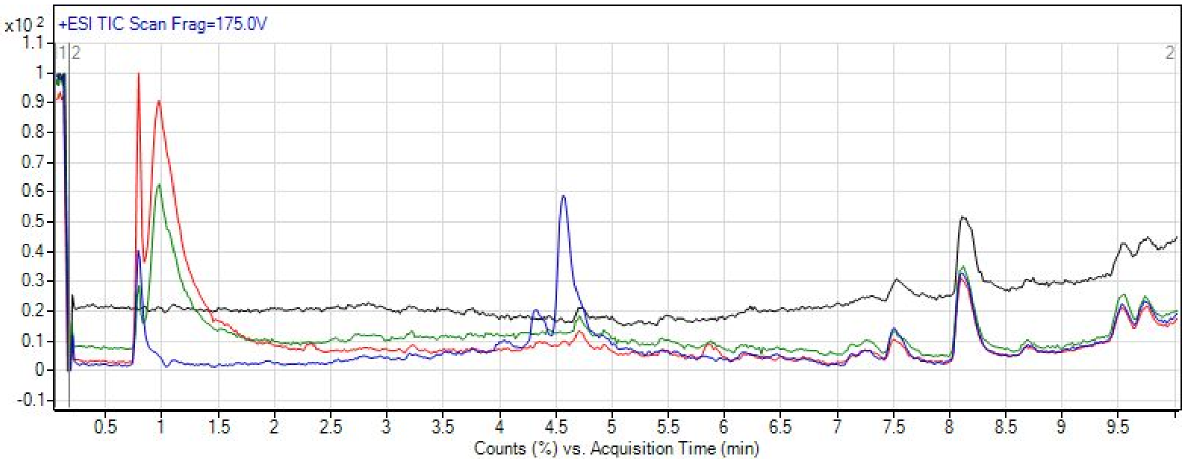

Figure S6.The total ion current chromatogram (TIC, ESI^{+}) for MB (blue line) and of samples obtained after photocatalytic degradation using KVO-20 (red line) and KVO-80 (green line) as a catalyst. The ion current chromatogram (TIC) for water was added as black line.

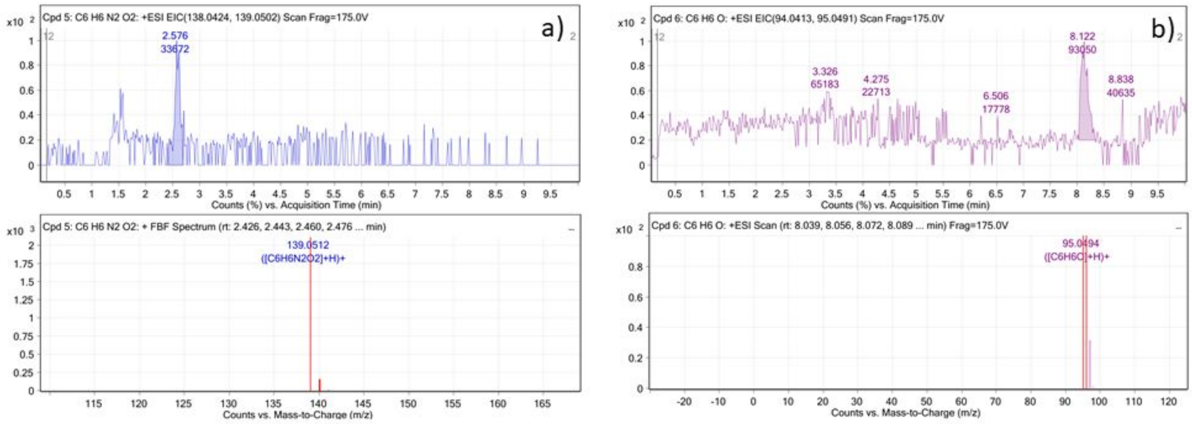

Figure S7.The chromatograms, and mass spectra for products identified after photocatalytic degradation of MB applying KVO-80 as catalyst.

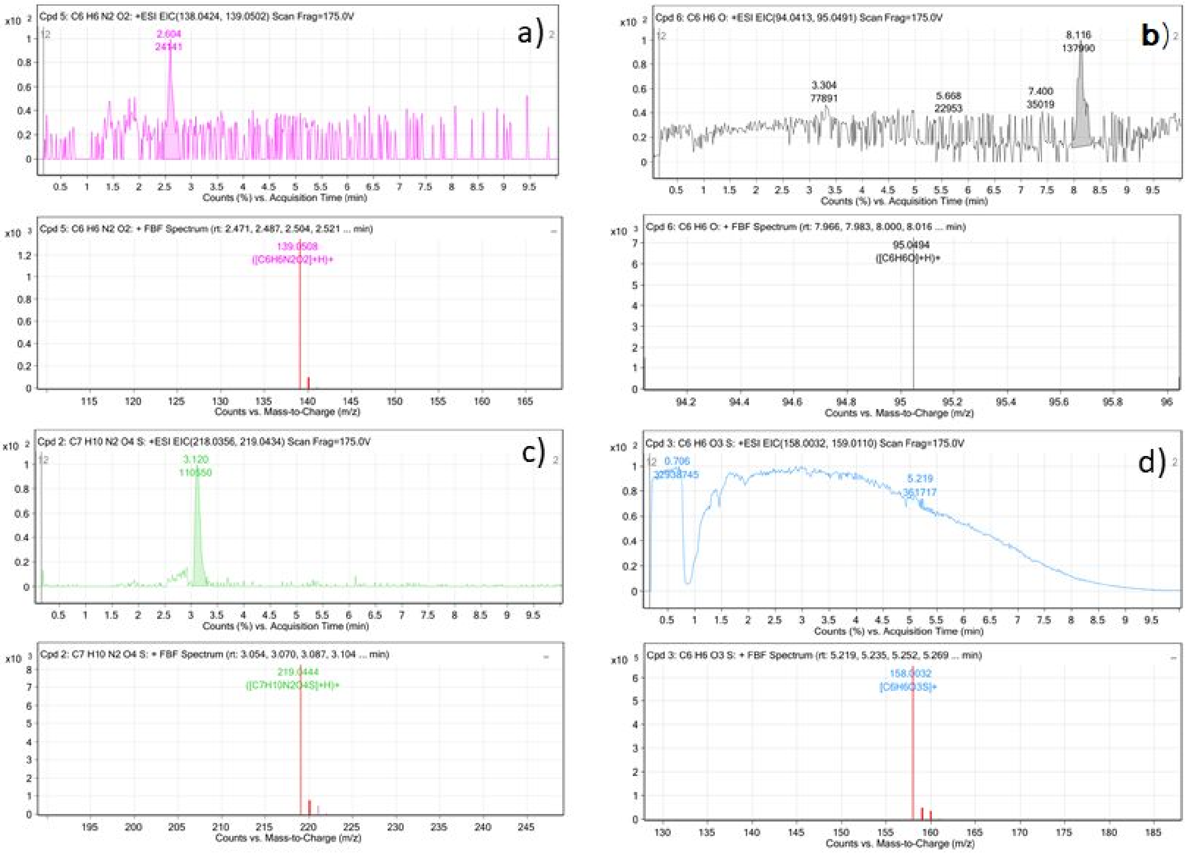

Figure S8.The chromatograms, and mass spectra for products identified after photocatalytic degradation of MB applying KVO-20 as catalyst.
